# Supplementary material for: FGF9 treatment reduces off-target chondrocytes from iPSC-derived kidney organoids
Source: NPJ Regen Med. 2025 Aug 30;10:41. doi: 10.1038/s41536-025-00428-9 (PMC12398568; doi:10.1038/s41536-025-00428-9)
Supplement: Supplementary file 1 — Supplementary information [file 41536_2025_428_MOESM1_ESM.pdf]

Supplementary Video 1: **Whole organoid staining with Alcian Blue revealed island of cartilage in kidney organoids at day 7+25.** Clearing of the soft tissues was performed after Alcian blue staining and revealed appearance of cartilage in day 7+25 kidney organoids from iPSC line LUMC0072iCTRL01.

Figure S1

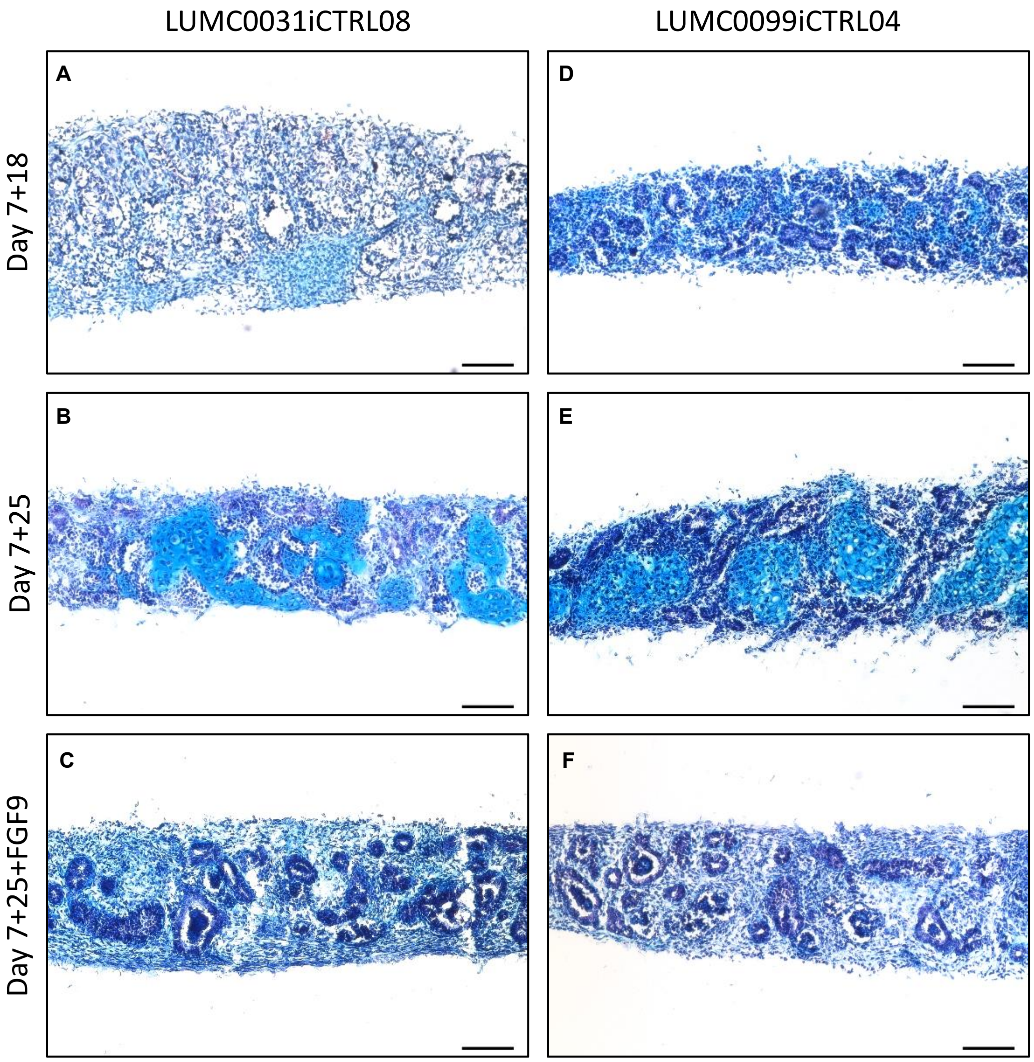

Figure S1: **FGF9-treated organoids present no cartilage at day 7+25.** Alcian blue staining revealed appearance of cartilage in day 7+25 kidney organoids from iPSC lines LUMC0031iCTRL08 (B) and LUMC0099iCTRL04 (E), and FGF9 treatment removes it (C and F). Scale bar: 100 μm.

Figure S2

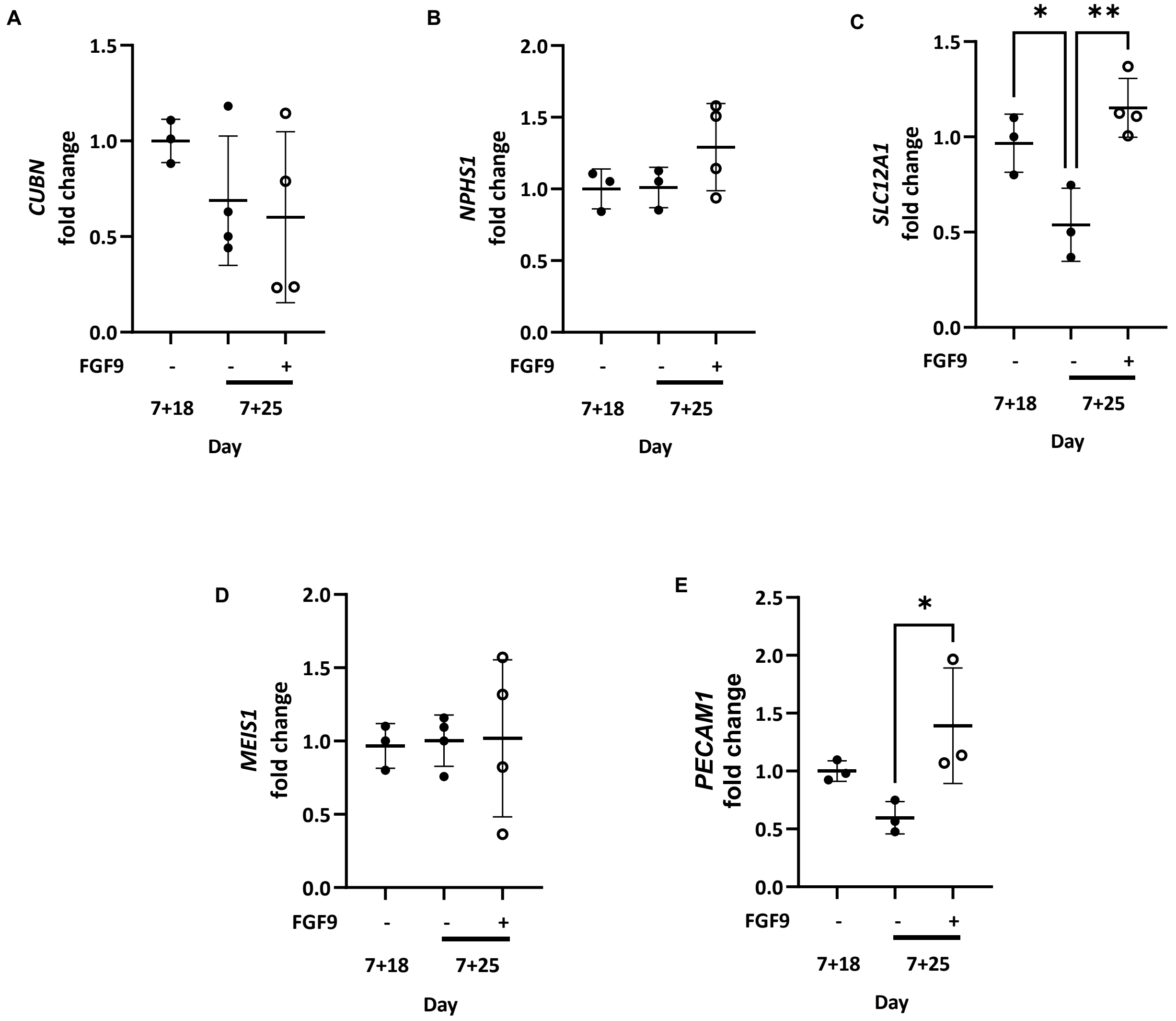

Figure S2: **FGF9 treatment does not negatively affect renal structures.** (A–D) Gene expression of markers of renal structures, *CUBN, NPHS1, SLC12A1* and stromal population *MEIS1* were assessed by qPCR and shown as –fold change compared to expression at day 7+18. *p < 0.05; **p < 0.01; from 3–4 samples. (E) FGF9 treatment (+) upregulates *PECAM1* expression compared to control (–) organoids at day 7+25. *PECAM1* expression assessed using qPCR shown as -fold change compared to expression in untreated organoids at day 7+18. *p < 0.05 from 3 samples.

Figure S3

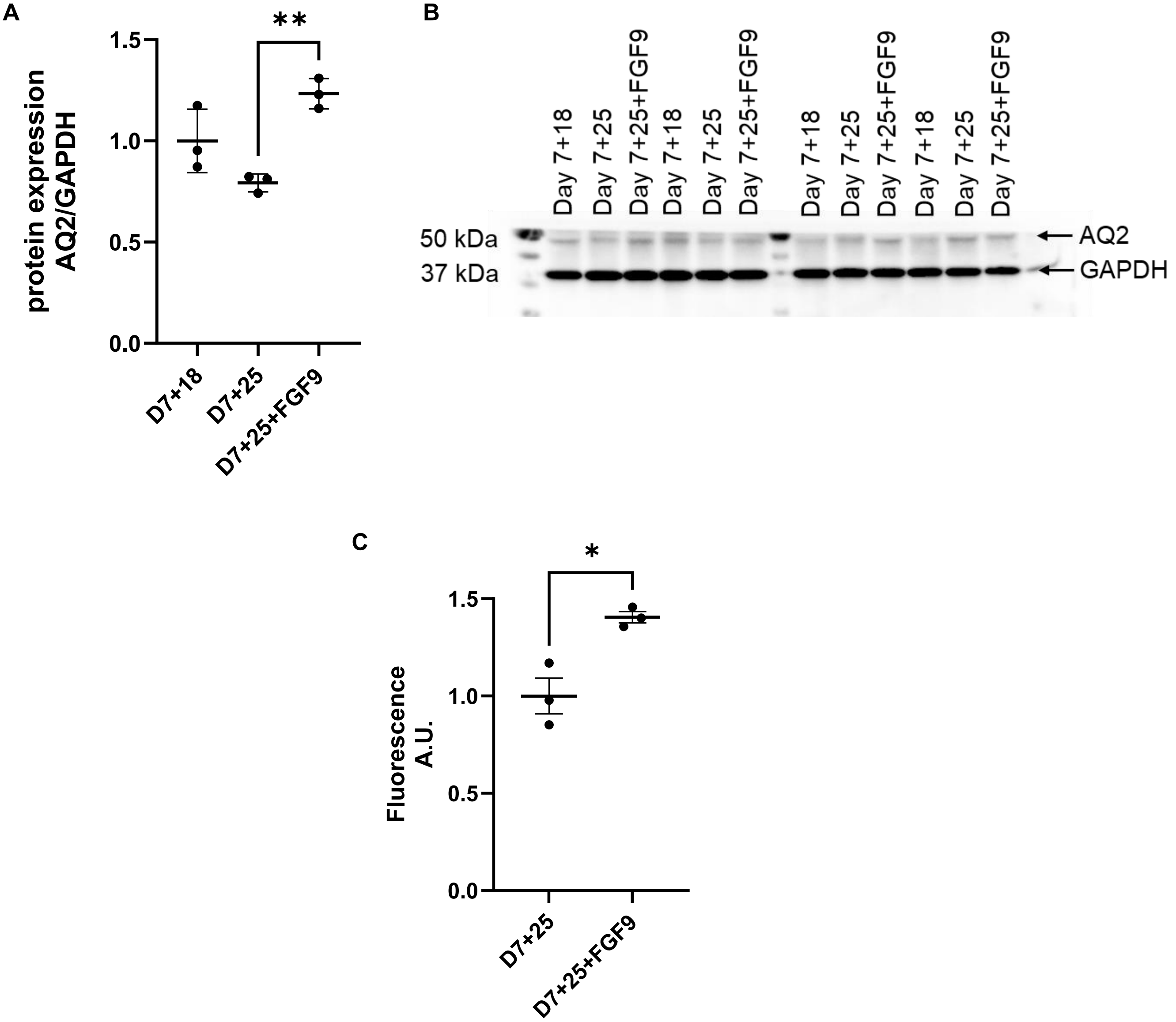

Figure S3: **FGF9 treatment affects AQ2 levels and BSA uptake.** (A) Quantification of AQ2 at days 7+18 and 7+25 showed no significant modulation in untreated organoids, while it was slightly increased by FGF9 treatment (7+25+FGF9). (B) Western immunoblotting showed lower levels of AQ2 in FGF9-treated (+) organoids compared to control (−) organoids at day 7+25. GADPH levels are shown as loading controls. Protein levels are expressed as -fold change relative to day 7+18. (C) Quantification of BSA fluorescence. Fluorescence is expressed as arbitrary unit (A.U.) relative to day 7+25. * $p < 0.05$ ** $p < 0.01$; from 3 samples each.

Figure S4

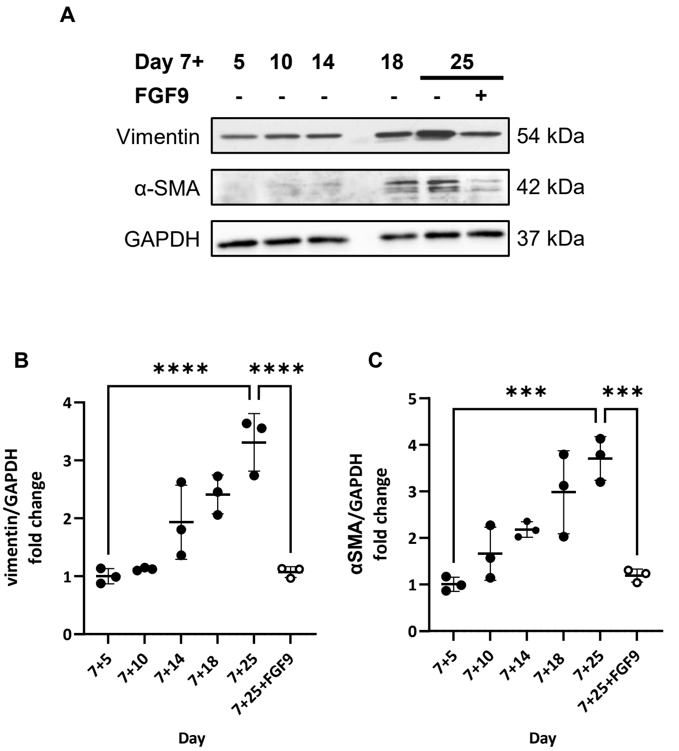

Figure S4: **FGF9-treated organoids express lower levels of EMT markers at day 7+25.** (A) Western immunoblotting showed lower levels of EMT markers vimentin and α-SMA in FGF9-treated (+) organoids compared to control (−) organoids at day 7+25. Notably, vimentin and α-SMA progressively increased from day 7+5, 7+10, 7+14, 7+18 and 7+25 in control organoids. GADPH levels are shown as loading controls. (B–C) Quantification of vimentin (B) and α-SMA (C) levels at day 7+5, 7+10, 7+14, 7+18 and 7+25 showed significant increases in untreated organoids, which were ameliorated by FGF9 treatment (7+25+FGF9). Protein levels are expressed as –fold change relative to day 7+5. *** p < 0.001; **** p < 0.0001 from 3 samples each.

Figure S5

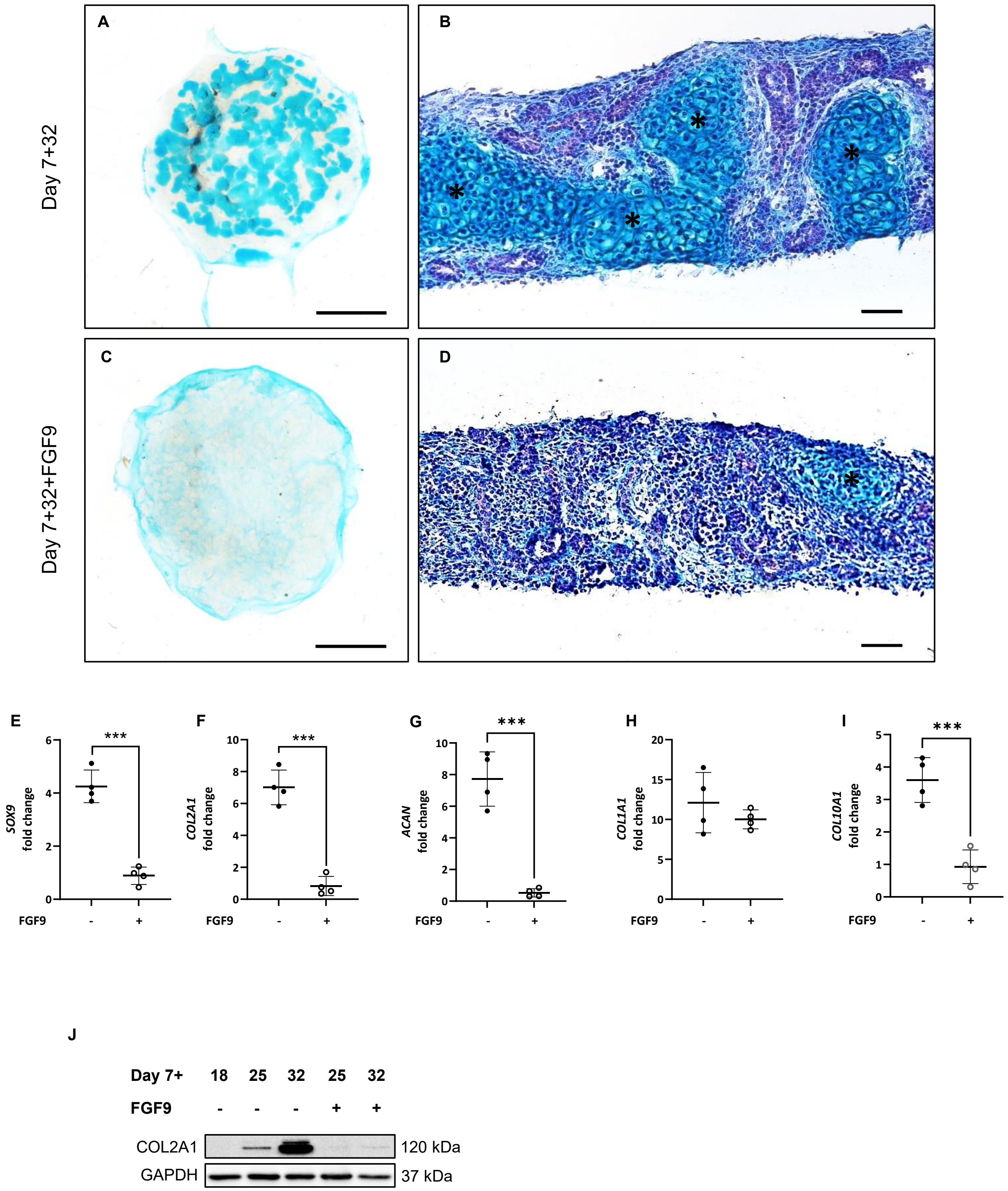
A
B
Day 7+32
C
D
Day 7+32+FGF9
E
SOX9 fold change
***
FGF9 - +
F
COL2A1 fold change
***
FGF9 - +
G
ACAN fold change
***
FGF9 - +
H
COL1A1 fold change
FGF9 - +
I
COL10A1 fold change
***
FGF9 - +
J
Day 7+ 18 25 32 25 32
FGF9 - - - + +
COL2A1 120 kDa
GAPDH 37 kDa

Figure S5: **FGF9 treatment delays the appearance of cartilage in iPSC-derived kidney organoids.** (A–D) Cartilage (asterisks) stained with Alcian blue in whole organoids (left images, scale bars represent 1000 μm) and on cryosections (right images, scale bars represent 50 μm) was less abundant with FGF9 treatment but the appearance of small islands of cartilage were visible (bottom row). (E–I) FGF9 treatment (+) significantly decreased four of five markers of chondrogenesis in kidney organoids at day 7+32 compared to control (−) organoids. Gene expression assessed by qPCR and shown as -fold change compared to expression in untreated organoids at day 7+18. ***$p < 0.001$ from 4 samples. (J) Western blotting COL2A1 protein in control (−) kidney organoids showed increased expression over time which was abrogated with FGF9 treatment (+). GADPH levels are shown as loading controls.

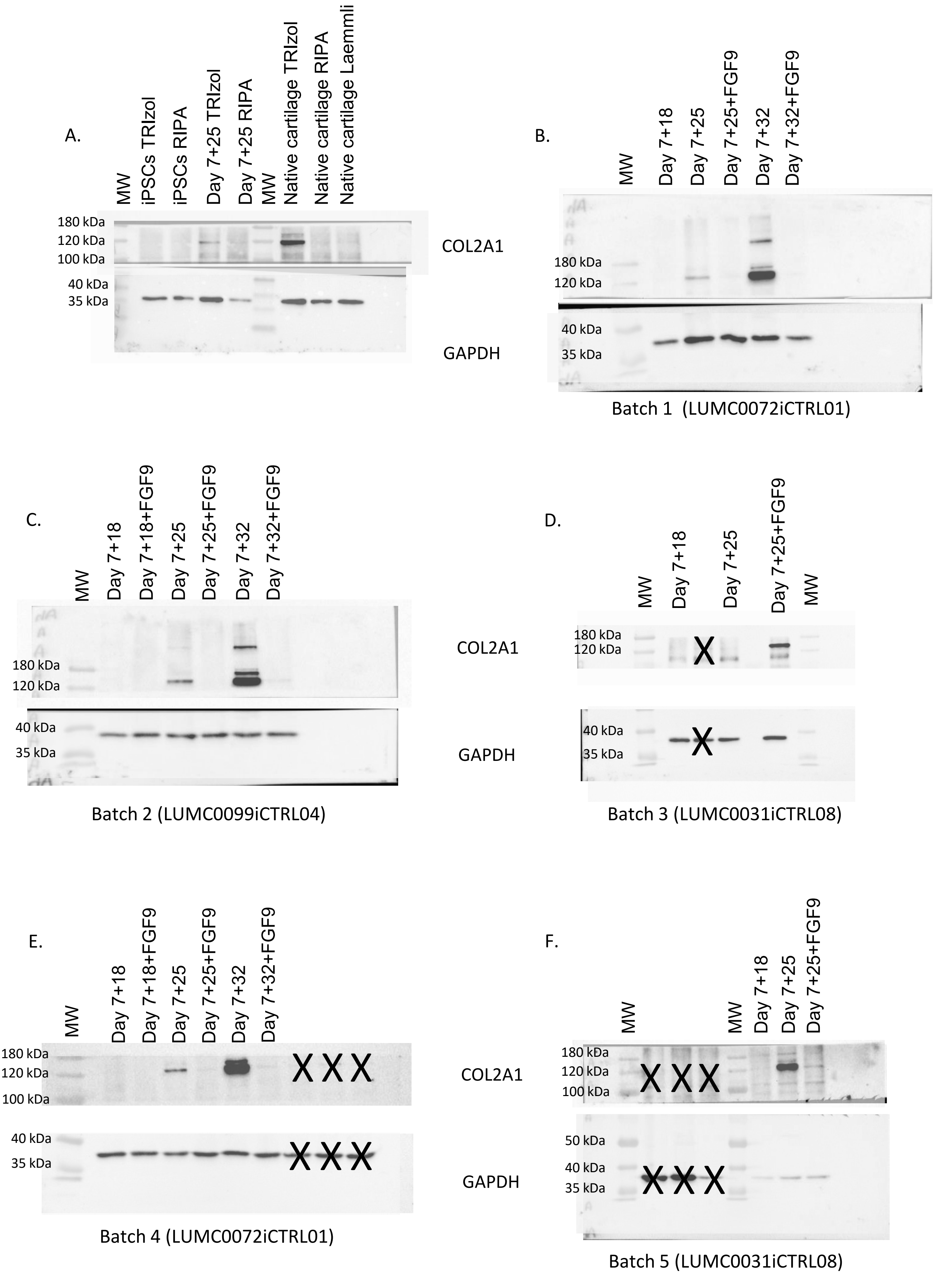

A.
MW
iPSCs TRIzol
iPSCs RIPA
Day 7+25 TRIzol
Day 7+25 RIPA
MW
Native cartilage TRIzol
Native cartilage RIPA
Native cartilage Laemmli
180 kDa
120 kDa
100 kDa
40 kDa
35 kDa
COL2A1
GAPDH
B.
MW
Day 7+18
Day 7+25
Day 7+25+FGF9
Day 7+32
Day 7+32+FGF9
180 kDa
120 kDa
40 kDa
35 kDa
Batch 1 (LUMC0072iCTRL01)
C.
MW
Day 7+18
Day 7+18+FGF9
Day 7+25
Day 7+25+FGF9
Day 7+32
Day 7+32+FGF9
180 kDa
120 kDa
40 kDa
35 kDa
Batch 2 (LUMC0099iCTRL04)
COL2A1
GAPDH
D.
MW
Day 7+18
Day 7+25
Day 7+25+FGF9
MW
180 kDa
120 kDa
40 kDa
35 kDa
Batch 3 (LUMC0031iCTRL08)
E.
MW
Day 7+18
Day 7+18+FGF9
Day 7+25
Day 7+25+FGF9
Day 7+32
Day 7+32+FGF9
180 kDa
120 kDa
100 kDa
40 kDa
35 kDa
Batch 4 (LUMC0072iCTRL01)
COL2A1
GAPDH
F.
MW
MW
Day 7+18
Day 7+25
Day 7+25+FGF9
180 kDa
120 kDa
100 kDa
50 kDa
40 kDa
35 kDa
Batch 5 (LUMC0031iCTRL08)

G.

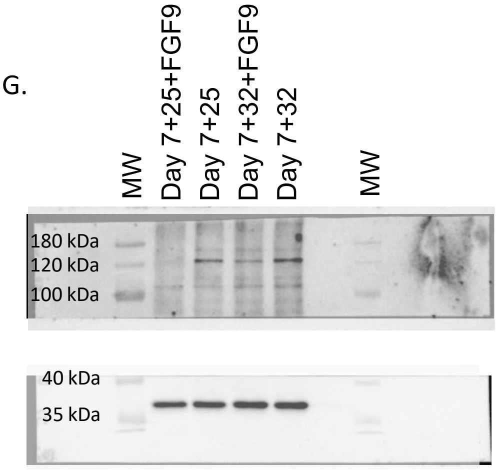

Batch 6 (LUMC0099iCTRL04)

H.

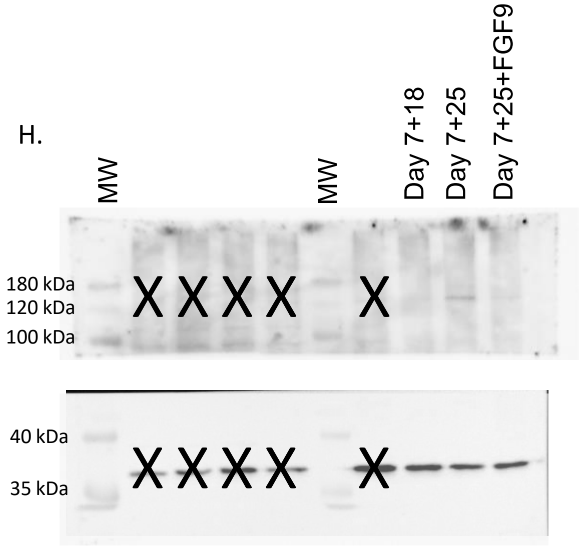

Batch 5 (LUMC0072iCTRL01)

I.

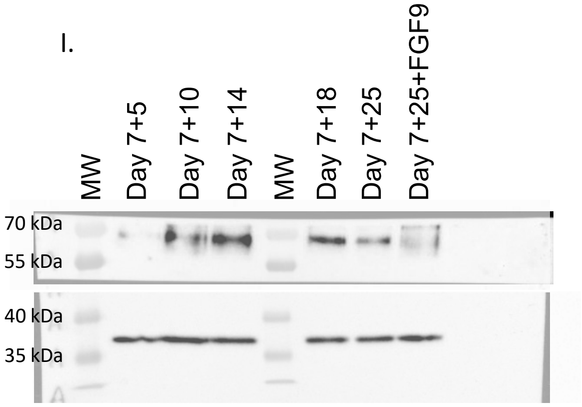

Batch 1 (LUMC0072iCTRL01)

J.

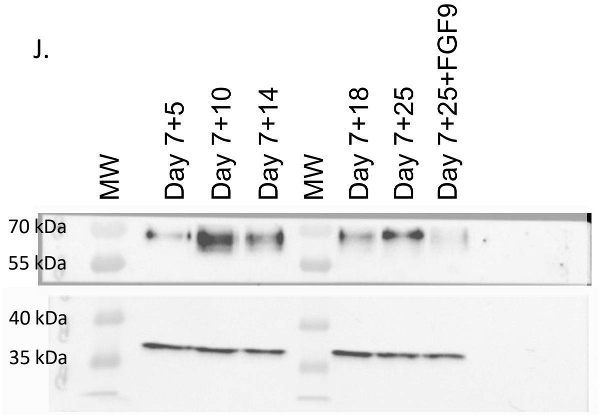

Batch 2 (LUMC0099iCTRL04)

K.

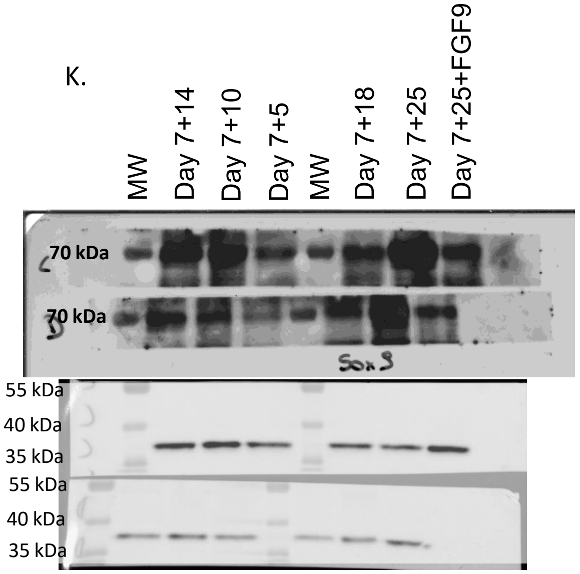

Batch 3 (LUMC0031iCTRL08)
Duplicate 1 and 2

L.

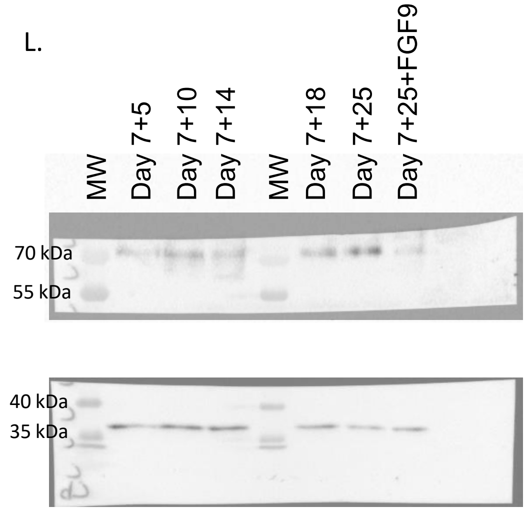

Batch 4 (LUMC0072iCTRL01)

Figure S6: Raw blots of cartilage markers. (A) Validation of COL2A1 antibody on negative (iPSCs) and positive (native human cartilage) controls and kidney organoids. Raw blots of COL2A1 (B-H) and SOX9 (I-L) in kidney organoids treated or not with FGF9 at different timepoints. Blots were imaged using ECL film or Chemidoc technology. When using Chemidoc, the imaging was performed using the "intense band" option and the background was automatically adjusted by the equipment to be white. MW= molecular weight (ladder)

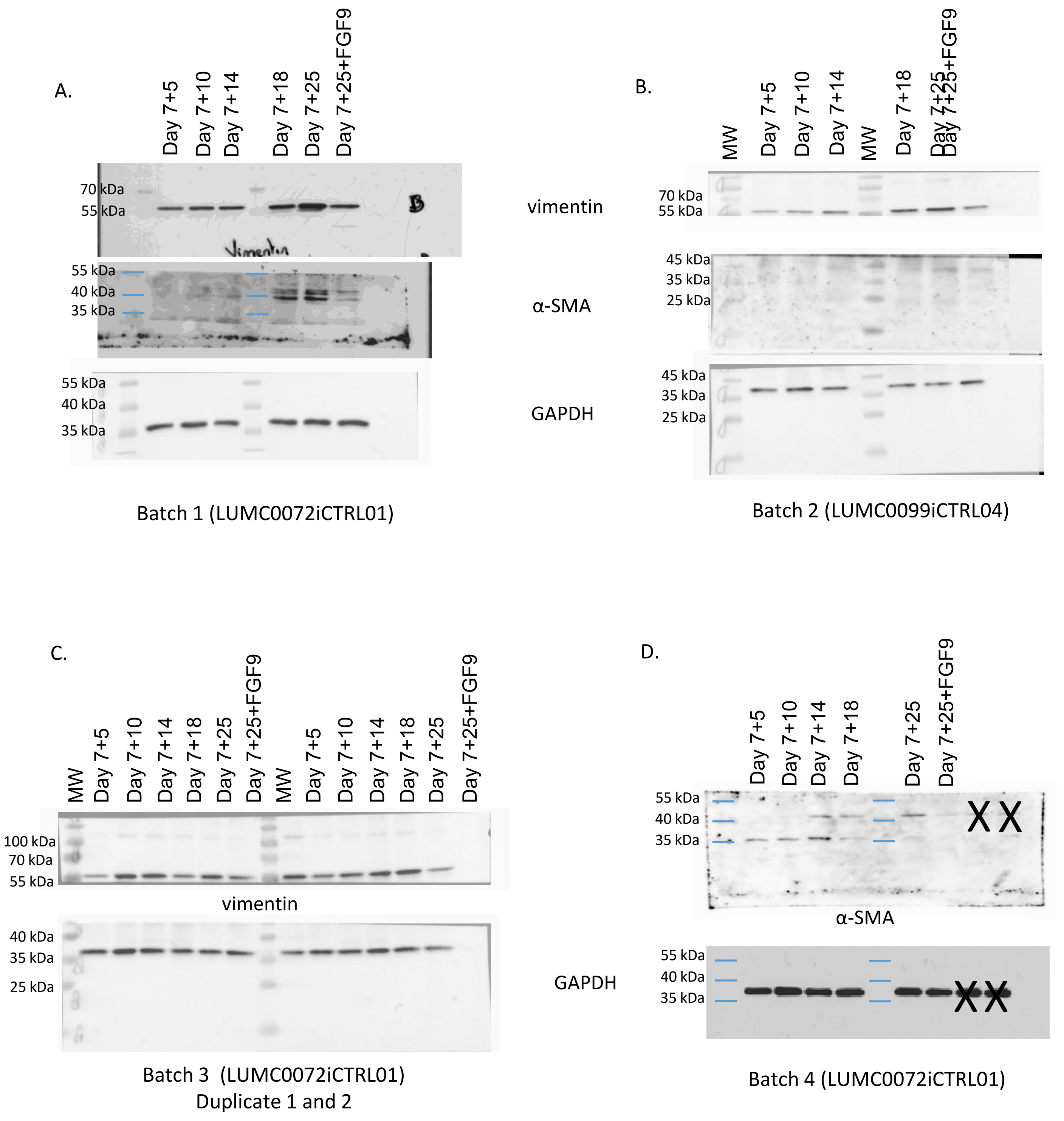

Figure S7: Raw blots of EMT markers. Raw blots of Vimentin (A-C) and a-SMA (A,B and D) in kidney organoids treated or not with FGF9 at different timepoints. Blots were imaged using ECL film or Chemidoc technology. When using Chemidoc, the imaging was performed using the “intense band” option and the background was automatically adjusted by the equipment to be white.

Supplementary Table 1: Primary and secondary antibodies used for immunofluorescence

**Primary antibodies**	**Species**	**Company**	**Reference**	**Dilution**
Nephrin 1	Sheep	R&D Systems	AF4269	1/300
E-cadherin	Mouse	Beckton Dickinson	BD 610182	1/300
SLC12A1	Rabbit	Sigma-Aldrich	HPA014967	1/300
MEIS1/2/3	Mouse	Santa Cruz Biotechnology	sc-101850	1/300
CD31	Mouse	Abcam	ab24590	1/300
Aquaporin 2	Mouse	Santa Cruz Biotechnology	sc-515770	1/300
Lotus lectin biotin conjugated (LTL)	/	Brunschwig	B-1325	1/300
**Secondary antibodies**		**Company**	**Reference**	**Dilution**
Alexa Fluor donkey anti-sheep 488		Thermo Fisher Scientific	A-11015	1/400
Alexa Fluor goat anti-mouse 568		Thermo Fisher Scientific	A-11031	1/400
Alexa Fluor goat anti-rabbit 568		Thermo Fisher Scientific	A-10042	1/400
Alexa Fluor goat anti-mouse 488		Thermo Fisher Scientific	A-11029	1/400
Alexa Fluor goat anti-sheep 647		Thermo Fisher Scientific	**A-21448**	1/400
Streptavidin Alexa Fluor 647 conjugate		Thermo Fisher Scientific	S-21374	1/1000

Supplementary Table 2: Primers used in qPCR

Primers	Sequences forward (F) and reverse (R) 5' to 3'
GAPDH	F: CTGGGCTACACTGAGCACC<br>R: AAGTGGTCGTTGAGGGCAATG
SOX9	F: GAGCCGAAAGCGGAGCTGGAA<br>R: ACAGCTGCCCGCTCCAAGTG
COL2A1	F: AACCAGATTGAGAGCATCCG<br>R: ACCTTCATGGCGTCCAAG
ACAN	F: TCGAGGACAGCGAGGCC<br>R: TCGAGGGTGTAGCGTGTAGAGA
COL1A1	F: CGGTGGTTTCTTGGTCGGT<br>R: GTGCGATGACGTGATCTGTGA
COL10A1	F: AAGAATGGCACCCCTGTAATGT<br>R: ACTCCCTGAAGCCTGATCCA
CUBN	F: TCCACCCGTTGAGTGTGTG<br>R: AGGAACCTAGAGTTGAGGAGC
NPHS1	F: TCACCGTGAATGTTCTGTTCC<br>R: AGTGTGGCTAAGGGATTACCC
SLC12A1	F: AGTGCCCAGTAATACCAATCGC<br>R: GCCTAAAGCTGATTCTGAGTCTT
MEIS	F: GGGCATGGATGGAGTAGGC<br>R: GGGTACTGATGCGAGTGCAG
PECAM1	F: AACAGTGTTGACATGAAGAGCC<br>R: TGTAAAACAGCACGTCATCCTT
